# The Cost of Delayed Transcatheter Aortic Valve Replacement

**DOI:** 10.1016/j.shj.2026.100822

**Published:** 2026-02-19

**Authors:** Bharat Rawlley, Harold L. Dauerman

**Affiliations:** Department of Medicine, University of Vermont Larner College of Medicine, Burlington, Vermont, USA

Both the 2025 European Society of Cardiology and the 2020 American College of Cardiology/American Heart Association guidelines recommend prompt aortic valve replacement for patients with symptomatic severe aortic stenosis (AS).^1^^,^^2^ In this issue of *Structural Heart*, Vemulapalli et al. evaluate the clinical and economic consequences of delayed transcatheter aortic valve replacement (TAVR) using a 90-day threshold from the diagnosis of symptomatic severe AS to valve intervention, consistent with established American Heart Association quality metrics.^3^^,^^4^

Using the Optum Market Clarity database, the authors analyzed 4,069 patients who underwent TAVR between July 2019 and June 2023. Of these, 2,051 (50.4%) underwent timely TAVR and 2,018 (49.6%) underwent delayed TAVR, defined as valve intervention performed more than 90 days after the diagnosis of symptomatic severe AS or performed on an urgent or emergent basis. Baseline demographics and overall comorbidity burden (assessed using the Elixhauser Comorbidity Index) were similar between the two groups. However, patients in the delayed TAVR group had higher Hospital Frailty Risk Scores than those treated in a timely manner (10.9 ± 9.4 vs. 8.5 ± 7.7, *p* < 0.01). The authors report that delayed TAVR was associated with higher rates of all-cause mortality, heart failure hospitalizations, and disabling stroke hospitalizations. Delayed treatment was associated with substantially higher downstream health care costs and higher hospitalization rates, with cardiac-related admissions accounting for a substantial proportion of this excess utilization. To examine the robustness of their findings, the authors performed a sensitivity analysis restricted to elective TAVR procedures. Delayed TAVR remained associated with a higher risk of the composite endpoint of all-cause mortality, heart failure hospitalization, and disabling stroke. In addition, the excess health care costs persisted among patients undergoing delayed TAVR compared with those treated in a timely manner.

The association between longer wait times for TAVR and worse clinical outcomes is well established in prior registry and trial analyses (Table 1).^5^, ^6^, ^7^, ^8^ Furthermore, this relationship has been examined in patients with both asymptomatic and symptomatic AS. In asymptomatic patients, the EARLY TAVR trial demonstrated that untreated severe AS is associated with progressive extravalvular cardiac remodeling as well as increased cardiovascular hospitalizations.^8^^,^^9^ Among patients with symptomatic severe AS, prior analyses from population-based registries in Canada have similarly shown adverse clinical consequences associated with longer wait times.^6^^,^^7^ However, an overview of the EARLY TAVR trial and prior registry analyses demonstrate a general consistency of wait time adverse events but a marked inconsistency in study design and endpoint assessment that limits conclusions (Table 1).

**Table 1:** Studies on delays to transcatheter aortic valve replacement (TAVR)

Author name (publication year)	Number (TAVR) and enrollment period	Study setting	TAVR wait times	Outcomes assessed
Vemulapalli et al.^3^ (2026)	N = 4069 2019-2023	Optum Market Clarity database (US)	≤ 90 d from diagnosis: 50.4% > 90 d from diagnosis or urgent/emergent: 49.6%	Delayed patients incurred $36,740 higher health care costs over 3 y ($182,470 vs. $145,730; *p* < 0.01), driven primarily by increased hospitalizations ($22,127 difference).
Srinivasan et al.^5^ (2023)	N = 831 2019-2022	Single Rural Health Network (Vermont)	≤ 30 d: 51.3% 31-90 d: 48.7%	Patients who required catheterization or percutaneous coronary intervention (PCI) before TAVR were significantly less likely to achieve TAVR within 30 d (OR 0.65, *p* = 0.01).
Albassam et al.^6^ (2020)	N = 3894 2012-2018	Ontario TAVR Population Based Registry (Ontario, Canada)	Median wait time 84 d	Wait list mortality for TAVR: 5.2% Heart failure hospitalization while on wait list for TAVR: 7.7%
Elbaz-Greener et al.^7^ (2018)	N = 2231 2010-2016	Ontario TAVR Population Based Registry (Ontario, Canada)	Median wait time 80 d	Wait list mortality and heart failure hospitalization at 80 d was 2% and 12%
Genereux P et al.^8^ (2024)	N = 455 TAVR N = 446 Clinical Surveillance	Randomized Trial at 75 centers in US and Canada	During 3.8 y median follow up, 87% of surveillance patients underwent TAVR	A strategy of early TAVR for asymptomatic AS superior to clinical surveillance for death, stroke or unplanned hospitalization

Abbreviations: AS, aortic stenosis; OR, odds ratio.

For example, although the absolute number of TAVR procedures in this Optum registry is higher than in prior analyses (Table 1), earlier studies were drawn from larger referral bases^6^ including population-based studies in Canada which are more likely to be generalizable. Second, 18% of patients (1,948 of 10,761 patients initially identified in the Optum Market Clarity data set with clinically significant AS) were excluded from the current analysis due to the absence of a recorded TAVR procedure within 2 years of the diagnosis of symptomatic severe AS. This may introduce bias by excluding patients with a high burden of comorbidities or patients whose procedures were not captured within the database. Third, clinical outcomes such as heart failure hospitalizations were assessed only after TAVR, which is in contrast to prior registry studies on the topic and also, the EARLY TAVR trial,^8^ which focused on events accruing during the waiting period before valve intervention. However, this analysis is novel in linking delayed TAVR to health care economic outcomes, uniquely integrating clinical endpoints with the financial implications of delayed valve intervention.

One important limitation of the current registry is that operational barriers to timely TAVR are not clearly defined. For example, although patients in the delayed TAVR group were more frail, the specific reasons for delay in these patients are unclear and may include intercurrent hospitalizations for noncardiac reasons, difficulties with travel, or concomitant dementia. In such patients, competing risks related to comorbid disease may be sufficiently high that outcomes and health care utilization remain elevated regardless of TAVR timing, thereby limiting the incremental benefit of earlier TAVR. Other operational factors that may influence time to TAVR include the need for coronary angiography before the procedure.^5^ However, the current data set does not capture operational metrics associated with delay, nor does it account for factors that could be examined in more granular analyses, such as travel time to the TAVR center.

Thus, the current study reinforces prior evidence that delays in TAVR for patients with severe AS are associated with adverse clinical consequences and uniquely integrates these findings with the health care economic costs of delayed valve intervention. Important unanswered questions remain and warrant further consideration. First, what operational policies could be introduced to reduce wait times for TAVR and limit patient exposure to delayed valve intervention? Second, how can access to care and the timing of intervention be optimized for patients with frailty? Third, can echocardiographic and imaging-based risk stratification be used to identify patients most likely to benefit from earlier intervention, an approach that may be particularly important for prioritization in resource-limited settings? Given the ever expanding volume of TAVR now for patients with symptomatic AS and the further increment that might occur with treatment of asymptomatic severe AS, system wide changes to improve timely access are critical to both improved clinical outcomes and cost efficient strategies.

## Funding

The authors have no funding to report.

## Disclosure Statement

Dr Dauerman is a consultant for 10.13039/100004374Medtronic and Laplace Interventional and has research grants from 10.13039/100008497Boston Scientific and 10.13039/100004374Medtronic. Dr Rawlley has no financial disclosures.
